# Optimization of the Mit1C real-time PCR assay using Bio-Rad CFX96 for the detection of *Cyclospora cayetanensis* in agricultural waters

**DOI:** 10.1371/journal.pone.0355957

**Published:** 2026-08-12

**Authors:** Khai Truong, Miely Suarez, Mauricio Durigan, Laura Ewing-Peeples, Mark C. Jenkins, Aminata Kilungo, Margarethe A. Cooper, Kerry K. Cooper, Gerardo U. Lopez

**Affiliations:** 1 School of Animal and Comparative Biomedical Sciences, The University of Arizona, Tucson, Arizona, United States of America; 2 Division of Food and Environmental Safety, Human Foods Program, Office of Laboratory Operations and Applied Science, Office of Applied Microbiology and Technology, U.S. Food and Drug Administration, Laurel, Maryland, United States of America; 3 Animal Parasitic Diseases Laboratory, Agriculture Research Service, U.S. Department of Agriculture, Beltsville, Maryland, United States of America; 4 BIO5 Institute, The University of Arizona, Tucson, Arizona, United State of America; University of Alcala Faculty of Medicine and Health Sciences: Universidad de Alcala Facultad de Medicina y Ciencias de la Salud, SPAIN

## Abstract

Increased cases of cyclosporiasis within the United States linked to domestically grown fresh produce highlight the need to identify sources of environmental contamination for *Cyclospora cayetanensis*. Recently, the Mit1C real-time PCR assay was validated by the U.S. FDA as the US-FDA’s BAM 19c for molecular detection of *C. cayetanensis* Cox3 gene in agricultural water using the now discontinued ABI-7500 platform. This study optimized the Mit1C real-time PCR assay on the Bio-Rad CFX96 platform for molecular detection of *C. cayetanensis* in environmental water. Using the Mit1AA synthetic fragment suspended in a 5 µg/mL salmon sperm carrier for standard curves, various combinations of cycling times, annealing temperatures, and primer/probe concentrations were optimized to achieve reproducible, sensitive, specific, and efficient amplification signal. Cross-reactivity was addressed with an exclusivity panel alongside an agricultural water positive control spiked with 200 *C. cayetanensis* oocysts and com-pared on both platforms. Results suggested only decreasing the annealing temperature (66°C), increasing cycles (42), and increasing cycle cutoff (Cq ≤ 40) to achieve performance comparable to the ABI-7500. Directly utilizing the ABI-7500 protocol for the Bio-Rad CFX96 platform diminished assay sensitivity and efficiency. We adapted the Mit1C real-time PCR protocol for the Bio-Rad CFX96 platform and suggest that instrument differences can have considerable impact on assay performance.

## Introduction

*Cyclospora cayetanensis* is a coccidian protozoan parasite and the causative agent of cyclosporiasis, a human-specific gastrointestinal disease emerging as a global public health concern [1]. In the United States, laboratory confirmed cases of domestically-acquired cyclosporiasis have significantly increased from hundreds to thousands annually since 2018 [2,3]. Reported illnesses and multi-state outbreaks are primarily associated with the consumption of fresh produce such as herbs, leafy greens, and berries [4]. Unlike most parasites, *C. cayetanensis* oocysts are not transmitted through direct fecal-oral contact, but require 1–2 weeks in the environment to sporulate before becoming infectious [5]. Protozoans shed from the feces of humans and animals can contaminate soil and water sources, therefore potentially contaminating downstream irrigation waters for produce intended for raw consumption [6–8]. Prior studies have detected *C. cayetanensis* in various types of waters, such as wastewater [7–9], surface water [8,10], and even drinking water [10–12]. Additionally, a systematic review estimated the global pooled prevalence of the parasite in water to be 6.9% and a prevalence in irrigation waters at 17.1% among six other types of water [13]. Therefore, agricultural waters should be examined as a potential vehicle for transmission of *C. cayetanensis* oocysts onto fresh produce.

A robust, sensitive, and specific method for the detection of *C. cayetanensis* is especially important for environmental samples, which are a complex matrix of various organisms and organic debris that can potentially make detection difficult. The traditional and standardized method for the molecular detection of *C. cayetanensis* in fresh produce and agricultural waters was a multi-laboratory validated quantitative real-time PCR assay targeting the 18S rRNA gene [14,15]. However, later reports of potential cross-reactivity from *in silico* and *in vitro* examination under different experimental conditions with closely related protozoans in the Eimeriidae family has led to a transition towards the new Mit1C real-time PCR assay targeting the mitochondrial *Cox3* gene developed by the United States Food and Drug Administration (FDA) [16–18]. Recently, the U.S. FDA adapted and released this protocol into the Bacteriological Analytical Manual as BAM Chapter 19c [19]. However, this Mit1C real-time PCR assay was only optimized on the Applied Biosystems 7500 (ABI-7500) real-time PCR platform, and robustness was clearly affected when compared to the Bio-Rad CFX96 [16]. As it is conceivable other laboratories may use a different platform besides the validated ABI-7500 [18], optimization may be required to ensure a consistently robust method for detection. Though maintaining the exact reagent setup seems sufficient to reproduce the validated Mit1C real-time PCR protocol, the impact of variations in real-time PCR instruments has yet to be determined.

In this study, we optimized the Mit1C real-time PCR assay for the Bio-Rad CFX96 real-time PCR platform to identify any necessary modifications from the ABI-7500 protocol described in the BAM Chapter 19c. To harmonize the molecular detection of *C. cayetanensis* across multiple laboratories, both real-time PCR protocols were compared to determine if performance is comparable.

## Materials and methods

### Internal amplification control

The exogenous and non-competitive internal amplification control (IAC) described by Deer et al. is a 200 bp Ultramer that was synthesized by Integrated DNA Technologies (IDT, Coralville, IA) and utilized in prior studies to monitor for matrix-associated inhibition during molecular detection in agricultural waters and fresh produce [2,6,15,20,21]. Each real-time PCR assay reaction contained the IAC Ultramer at a desired concentration of 10^4^ copies with IAC primer and probe concentrations both at 250 nM [6,16].

### Synthetic Mit1AA positive control

The Mit1AA as described by Balan et al. (2023) is a commercially synthesized 245 bp DNA gBlocks (IDT) based on sequence data from the targeted *C. cayetanensis* mitochondrial *Cox3* gene region [16]. The Mit1AA gBlock contains a traceable double adenine mutation distinguishable from wild-type DNA to monitor for internal laboratory contamination and was utilized as a positive control for the Mit1C real-time PCR optimization assay. The positive control was suspended in 10 mM Tris and 0.1 mM ethylenediaminetetraacetic acid (EDTA) buffer (IDT) containing 5 µg/mL salmon sperm DNA (Sigma Aldrich, Inc., St. Louis, MO) as a carrier. Serial dilutions were performed to obtain working solutions of the positive control, where solutions with at least 1000 copies were stored at −20°C for no more than 2 days while the 100 and 10 copy dilutions were prepared fresh.

### Optimization of a real-time PCR assay

The main workflow of the real-time PCR optimization assay is described by Durigan et al. [6], with Mit1C primer and probe concentrations of 600nM and 300nM, respectively, and an annealing temperature of 67°C set as baseline at the start of the experiment. The optimization assay began on the Bio-Rad CFX96 (Bio-Rad, Hercules, CA) real-time PCR platform by testing three PCR cycling conditions summarized in Table 1, each differing in time for the denaturation as well as annealing and extension steps. The standard and fast cycling protocols were based on manufacturer’s recommendations, while the slow cycling protocol was chosen for increased target amplification. The denaturation temperature was kept constant at 95°C. Fluorescence reading was active during the annealing/extension step. Six annealing temperatures ranging from 63°C to 68°C in one-degree increments were evaluated, where the highest temperature was preferred to increase the stringency of the assay. Eight combinations of the Mit1C primer (100 nM, 200 nM, 450 nM, and 600 nM) and probe (250 nM and 300 nM) concentrations were also evaluated. All 20 µL real-time PCR assays were performed with the 2X PrimeTime Gene Expression Master Mix (no ROX; IDT) and a standard yielding 1000, 100, and 10 copies of the positive control. The 1000 and 100 copies dilutions were performed in triplicates, while the 10 copies dilution was performed in six replicates to increase robustness. PCR cycling, annealing temperature, and primer/probe concentration optimizations were performed in technical triplicates. A no template control (nuclease-free water) and negative control (salmon sperm DNA) in triplicates were included in each assay. All assays were performed in a AirClean 600 PCR Workstation to minimize contamination. Data analysis of real-time PCR data was conducted on the Bio-Rad CFX Manager (version 3.1) software with manual threshold at 200 relative fluorescence units (RFU) and baseline cycles auto calculated. The optimal reaction protocol was determined by a lower cycle threshold (Cq), linearity of R^2^ ≥ 0.98, and PCR efficiency of 80% − 120 [22]. The optimization assay was an iterative process, where each PCR protocol was tested across a range of other amplification conditions to determine the best overall combination.

**Table 1:** Testing three Real-time PCR cycling protocols that varied by cycle duration.

Cycling Protocol	Cycles (40x)	Duration
Fast	Initial Denaturation	3 min
	Denaturation	5 sec
	Anneal/Ext	30 sec
Standard	Initial Denaturation	3 min
	Denaturation	15 sec
	Anneal/Ext	1 min
Slow	Initial Denaturation	3 min
	Denaturation	15 sec
	Anneal/Ext	1.5 min

### Linearity, efficiency, and sensitivity

The linearity and PCR efficiency of the candidate real-time PCR protocol for the Bio-Rad CFX96 was assessed with a ten-fold serial dilution (10^5^–10^1^ copies) in twelve replicates and in technical triplicates. To determine impact on robustness, the same performance metrics for the ABI-7500 were provided by the FDA and generated under conditions as described by Durigan et al. [6]. Sensitivity was determined with a serial dilution (20, 10, 5, 2, 1, and 0.1 copies) as recommended by the Quodata web service (Last accessed February 19, 2025) to calculate the LOD_95%_ [6,16,23]. A no-template control (nuclease-free water) and negative control (salmon sperm DNA only) in triplicates was included in all assays.

### Exclusivity panel

Concerns of cross-reactivity were addressed with an exclusivity panel consisting of *Eimeria tenella*, *Eimeria maxima*, *Eimeria acervulina*, *Cryptosporidium parvum*, *Blastocystis hominis*, *Giardia intestinalis*, *Plasmodium falciparum*, *Cryptosporidium hominis*, *Plasmodium malariae*, *Toxoplasma gondii*, *Neospora caninum*, *Trypanosoma cruzi*, and *Plasmodium vivax* genomic DNA. DNA from agricultural water spiked with 200 *C. cayetanensis* oocysts received from the FDA were used as a positive control, alongside a no-template control. The real-time PCR assay was performed in triplicates with the determined optimized real-time PCR protocol and used 2 µL of template DNA to reach a desired concentration of 2 ng for other parasites. DNA extracts of *Eimeria* species and other parasites were kindly provided by the United States Department of Agriculture (USDA) and the FDA, respectively.

### Real-time PCR protocol and detecting *C. cayetanensis*

Based on initial data from this study indicating 66°C as the ideal temperature, the exclusivity panel was performed at 66°C and 67°C. Since the reference Mit1C qPCR protocol by Durigan et al. uses an annealing temperature of 67°C [6], this assay was performed to determine whether this reduction in annealing temperature would compromise specificity, and whether the assay could detect the DNA of the 200 *C. cayetanensis* oocyst positive control as previously mentioned.

## Results

### Optimized real-time PCR conditions for Bio-Rad CFX96

The optimized real-time PCR condition used the standard cycling parameters beginning with an initial denaturation at 95°C for 3 min followed by 42 cycles of denaturation (95°C; 15 sec) and annealing/extension (66°C; 1 min). The optimal primer/probe concentrations were determined to be 600 nM/300 nM, respectively. The efficiency and linearity of the real-time PCR protocol for the Bio-Rad CFX96 was consistently within acceptable ranges, indicating averages: PCR efficiency = 94.8%, R^2^ = 0.994, slope = −3.455, and Y-int = 40.21 that supported a theoretical cut-off value of Cq ≤ 40. Accordingly, the duration of the Mit1C qPCR assay was extended to 42 cycles to allow sufficient time for detection. Manually setting the baseline threshold to 200 relative fluorescence units (RFU) was determined to adequately exclude background noise whilst not compromising PCR efficiency and linearity. When comparing the optimized Mit1C real-time PCR protocols for the Bio-Rad CFX96 and ABI-7500 platforms, the only difference was an increase in cycles and Cq cutoff value, and a decrease in annealing temperature for the former (Table 2).

**Table 2:** Summary and comparison of the Mit1C real-time PCR assay on the Bio-Rad CFX96 and ABI-7500 real-time PCR platforms.

Real-time PCR Conditions	Bio-Rad CFX96	ABI-7500*
Initial Denaturation	95°C; 3 min	95°C; 3 min
Denaturation	95°C; 15 sec	95°C; 15 sec
Annealing/Extension	**66°C**; 1 min	**67°C**; 1 min
Mit1C Primer	600 nM	600 nM
Mit1C Probe	300 nM	300 nM
IAC Primer	250 nM	250 nM
IAC Probe	250 nM	250 nM
IAC Target	1.00E + 04 copies	1.00E + 04 copies
Cycles	**42**	**40**
Cq Cutoff	**40**	**38**
Efficiency	94.8%	96.1%
Linearity	0.99	0.99
Y-intercept	40.21	37.27

Differences in protocols are in bold.

*Data from Durigan et al. (2024).

### Impact on robustness

**Fig 1:**
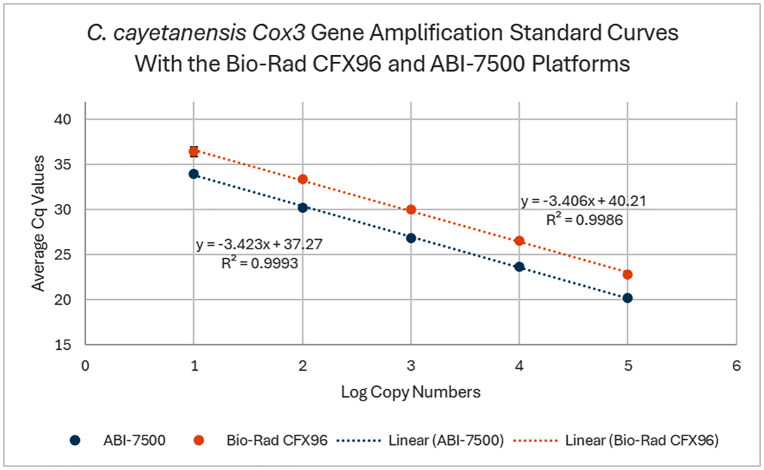
Comparison of standard curves for the detection of the *C. cayetanensis Cox3* gene using the Bio-Rad CFX96 and ABI-7500 real-time PCR platforms.

A side-by-side comparison of standard curves for the Bio-Rad CFX96 and ABI-7500 indicated higher average Cq values and Y-int for the Bio-Rad CFX96 (Fig 1). The standard curve for the Bio-Rad CFX96 indicated a Y-int = 40.21, while the standard curve for the ABI-7500 indicated a Y-int = 37.27. This is consistent with increasing the theoretical cutoff for the Bio-Rad CFX96 to Cq ≤ 40 as opposed to the ABI-7500 with a theoretical cutoff of Cq ≤ 38. Accordingly, the efficiencies were 96.7% for the Bio-Rad CFX and 96.01% for the ABI-7500, which were consistently within acceptable ranges. The linearity of both standard curves was very similar (R ≈ 0.99) and within acceptable ranges. Only no amplification was determined for the NTC, and amplification of the IAC in all reaction wells indicated no inhibition of the assay, as expected (Table 3).

**Table 3:** Side-by-side comparison of standard curve data from the Bio-Rad CFX96 and ABI-7500 real-time PCR platforms.

	Bio-Rad CFX96			ABI-7500		
Copy Number	*Cox3*		IAC	*Cox3*		IAC
	No. Positive Replicates	Mean Cq ± SD	Mean Cq ± SD	No. Positive Replicates	Mean Cq ± SD	Mean Cq ± SD
10^5^	12/12	22.82 ± 0.16	25.26 ± 0.11	3/3	20.21 ± 0.11	26.12 ± 0.03
10^4^	12/12	26.56 ± 0.14	25.14 ± 0.12	3/3	23.67 ± 0.10	26.17 ± 0.05
10^3^	12/12	30.07 ± 0.08	25.10 ± 0.04	3/3	26.87 ± 0.09	26.19 ± 0.03
10^2^	12/12	33.36 ± 0.24	25.08 ± 0.05	3/3	30.28 ± 0.16	26.13 ± 0.01
10^1^	12/12	36.45 ± 0.56	25.07 ± 0.07	3/3	34.02 ± 0.25	26.10 ± 0.03
NTC^*^	0/12	UND*	25.10 ± 0.06	0/3	UND^**^	26.16 ± 0.01

* No Template Control.

** UND: Undetermined (not detected).

### Impact on sensitivity and specificity

The Quodata web service indicated a theoretical LOD_95%_ of 3.64 copies (95% CI: [2.31, 5.72]) under ideal conditions (S1 Table). Only the *C. cayetanensis* DNA extract was amplified in all three replicates during the exclusivity panel with no indication of any latent off-target amplification below the baseline threshold (Table 4). Additionally, 66°C was further confirmed as the ideal annealing temperature as increasing to 67°C on the Bio-Rad CFX96 did not result in detectable amplification of the 200 *C. cayetanensis* oocysts DNA extract in agricultural water (Table 4).

**Table 4:** Exclusivity panel for the Mit1C real-time PCR assay optimized for the Bio-Rad CFX96 (66°C) and comparison to real-time PCR protocol for the ABI-7500 platform (67°C).

	Average Cq			
	66°C		67°C	
**Organism**	**Mit1C Target**	**IAC Target**	**Mit1C Target**	**IAC Target**
^*^ *Cyclospora cayetanensis*	35.06 ± 0.38	24.94 ± 0.05	UND	24.99 ± 0.02
*Eimeria tenella*	UND^**^	25.82 ± 0.17	UND	26.40 ± 0.05
*Eimeria maxima*	UND	25.65 ± 0.06	UND	26.32 ± 0.05
*Eimeria acervulina*	UND	25.53 ± 0.03	UND	26.27 ± 0.03
*Cryptosporidium parvum*	UND	25.03 ± 0.12	UND	25.08 ± 0.12
*Cryptosporidium hominis*	UND	24.90 ± 0.17	UND	24.96 ± 0.10
*Giardia intestinalis*	UND	24.80 ± 0.04	UND	24.92 ± 0.06
*Plasmodium falciparum*	UND	24.86 ± 0.11	UND	24.93 ± 0.10
*Plasmodium malariae*	UND	24.91 ± 0.02	UND	24.87 ± 0.05
*Plasmodium vivax*	UND	24.87 ± 0.08	UND	24.90 ± 0.07
*Blastocystis hominis*	UND	24.92 ± 0.16	UND	24.96 ± 0.14
*Toxoplasma gondii*	UND	24.85 ± 0.05	UND	24.89 ± 0.09
*Neospora caninum*	UND	24.86 ± 0.13	UND	24.94 ± 0.05
*Trypanosoma cruzi*	UND	24.92 ± 0.02	UND	24.95 ± 0.05

* 200 *C. cayetanensis* oocysts DNA extract in agricultural water.

** UND: Undetermined (not detected).

## Discussion

Standardization in pathogen detection methods are important for reliable and consistent reporting, which is especially needed to identify sources of contamination for emerging diseases. The specificity of the 18S rRNA real-time PCR assay has raised concerns about the potential detection of nontargeted organisms since *in silico* data showed that the primers targeting the *C. cayetanensis* 18S rRNA gene share strong sequence similarity to 18S rRNA genes of other parasites [6]. The inclusion of new datasets of mitochondrial genomic data enabled the development of detection assays based on the mitochondrial genome [6,16,24]. However, the FDA-validated method, known as BAM 19c, requires a specific set of reagents and platforms which might not be widely available. Adapting the protocol for more common platforms is therefore essential to facilitate broader research and testing capabilities. Development of a real-time PCR assay with improved specificity and comparable sensitivity to the original method should require optimization prior to validation for streamlined identification of target pathogens. This study demonstrates that choices in real-time PCR platforms regardless of identical reagent setup can have drastic changes in the Mit1C real-time PCR assay results, and potentially other molecular detection assays. As an obligate intracellular parasite incapable of reproducing freely in the environment, it is expected for *C. cayetanensis* to exist in very low concentrations in environmental samples [25]. Directly utilizing the ABI-7500 platform optimized Mit1C real-time PCR protocol for the Bio-Rad CFX96 platform would significantly diminish the assay’s sensitivity (Table 4), rendering it incapable of detecting environmentally relevant concentrations of *C. cayetanensis*. Consequently, false negative data is to be expected.

The robustness of the Mit1C real-time PCR assay was clearly affected by choice in platform, where lower Cq values were generated by the ABI-7500 platform (Fig 1). This variability was previously reported by Balan et al. [16], which highlights the need to optimize the protocol for the Bio-Rad CFX96. Accordingly, slight optimizations to the Mit1C real-time PCR assay for the Bio-Rad CFX96 platform returned performance comparable to the ABI-7500 platform. The original Mit1C real-time PCR method developed by Balan et al. (2023) performed with a theoretical LOD_95%_ within the 95% confidence interval reported in this study [16]. Notably, achieving a comparable level of sensitivity was primarily facilitated by a slight reduction in the annealing temperature for the Bio-Rad CFX96 platform without resulting in cross-reactivity, despite prior reports indicating that increased annealing temperatures enhance specificity [6]. In this case, a one-degree difference (67°C – 66°C) had a substantial effect on the sensitivity, where the Mit1C real-time PCR assay could not detect as high as 200 oocysts at 67°C (Table 4). Additionally, increasing the PCR cycle and threshold to 40 and 42 cycles, respectively, was also beneficial during optimization as it would allow accurate and timely detection of low target copy numbers, which is to be expected when screening environmental samples (S1 Table). Although other important parameters such as PCR cycle duration and primer/probe concentrations affect target yield and primer-template binding, we did not observe any necessary modifications when optimizing the Mit1C real-time PCR for the Bio-Rad CFX96. Overall, it is clear that variations in thermocycler performance can affect certain optimization parameters, which should be addressed for standardized testing. However, as this study primarily utilizes synthetic Mit1AA gBlocks in salmon sperm DNA, further evaluation of spiked environmental waters is needed to estimate sensitivity.

## Conclusions

This study suggests that instrumental variations can impact the Mit1C real-time PCR assay’s performance with regards to the *C. cayetanensis* mitochondrial *Cox3* gene. As it is conceivable for laboratories to utilize various other real-time PCR platforms for detecting *C. cayetanensis*, this study encourages optimization prior to large-scaled sample testing with the Mit1C real-time PCR assay.

## Supporting information

S1 Table Sensitivity of the Mit1C real-time PCR assay using Mit1AA synthetic fragments. (DOCX)

S1 File Supplemental Mit1C Optimization Data. (XLSX)

## References

[1] Almeria S, Chacin-Bonilla L, Maloney JG, Santin M. *Cyclospora cayetanensis*: a perspective (2020–2023) with emphasis on epidemiology and detection methods. Microorganisms. 2023;11(9):2171. doi: 10.3390/microorganisms11092171 37764015 PMC10536660

[2] Durigan M, Murphy HR, da Silva AJ. Dead-End ultrafiltration and DNA-based methods for detection of *Cyclospora cayetanensis* in agricultural water. Appl Environ Microbiol. 2020;86(23):e01595-20. doi: 10.1128/AEM.01595-20 32948525 PMC7657621

[3] U.S. Centers for Disease Control and Prevention. Parasites – Cyclosporiasis (*Cyclospora Infection*). https://www.cdc.gov/parasites/cyclosporiasis/outbreaks/index.html. Accessed 2024 February 5.

[4] Ortega YR, Sanchez R. Update on *Cyclospora cayetanensis*, a food-borne and waterborne parasite. Clin Microbiol Rev. 2010;23(1):218–34. doi: 10.1128/CMR.00026-09 20065331 PMC2806662

[5] Almeria S, Cinar HN, Dubey JP. *Cyclospora cayetanensis* and cyclosporiasis: an update. Microorganisms. 2019;7(9). doi: 10.3390/microorganisms7090317 PMC6780905 31487898

[6] Durigan M, Ewing-Peeples L, Almeria S, Balan KV, Grocholl J, Irizawa S, et al. Detection of cyclospora cayetanensis in food and water samples: optimized protocols for specific and sensitive molecular methods from a regulatory agency perspective. J Food Prot. 2024;87(7):100291. doi: 10.1016/j.jfp.2024.100291 38701974

[7] Kitajima M, Haramoto E, Iker BC, Gerba CP. Occurrence of *Cryptosporidium*, *Giardia*, and *Cyclospora* in influent and effluent water at wastewater treatment plants in Arizona. Sci Total Environ. 2014;484:129–36. doi: 10.1016/j.scitotenv.2014.03.036 24695096

[8] Plutzer J, Karanis P. Neglected waterborne parasitic protozoa and their detection in water. Water Research. 2016;101:318–32. doi: 10.1016/j.watres.2016.05.085 27281375

[9] Giangaspero A, Marangi M, Koehler AV, Papini R, Normanno G, Lacasella V, et al. Molecular detection of *Cyclospora* in water, soil, vegetables and humans in southern Italy signals a need for improved monitoring by health authorities. Int J Food Microbiol. 2015;211:95–100. doi: 10.1016/j.ijfoodmicro.2015.07.002 26188495

[10] Siwila J, Mwaba F, Chidumayo N, Mubanga C. Food and waterborne protozoan parasites: The African perspective. Food Waterborne Parasitol. 2020;20:e00088. doi: 10.1016/j.fawpar.2020.e00088 32995582 PMC7502820

[11] Dowd SE, John D, Eliopolus J, Gerba CP, Naranjo J, Klein R, et al. Confirmed detection of *Cyclospora cayetanesis*, *Encephalitozoon intestinalis* and *Cryptosporidium parvum* in water used for drinking. J Water Health. 2003;1(3):117–23. doi: 10.2166/wh.2003.0014 15384722

[12] Giangaspero A, Marangi M, Arace E. *Cyclospora cayetanensis* travels in tap water on Italian trains. J Water Health. 2015;13(1):210–6. doi: 10.2166/wh.2014.093 25719480

[13] Naganathan T, O’Connor A, Sargeant JM, Shapiro K, Totton S, Winder C, et al. The prevalence of *Cyclospora cayetanensis* in water: a systematic review and meta-analysis. Epidemiol Infect. 2021;150. doi: 10.1017/s0950268821002521

[14] U.S. Food and Drug Administration. BAM 19c: Dead-end ultrafiltration for the detection of Cyclospora cayetanensis from agricultural water. 2024. https://www.fda.gov/food/laboratory-methods-food/bam-19c-dead-end-ultrafiltration-detection-Cyclospora-cayetanensis-agricultural-water

[15] Murphy HR, Cinar HN, Gopinath G, Noe KE, Chatman LD, Miranda NE, et al. Interlaboratory validation of an improved method for detection of *Cyclospora cayetanensis* in produce using a real-time PCR assay. Food Microbiol. 2018;69:170–8. doi: 10.1016/j.fm.2017.08.008 28941898

[16] Balan KV, Mammel M, Lipman D, Babu U, Harrison LM, Almeria S, et al. Development and single laboratory evaluation of a refined and specific real-time PCR Detection Method, Using Mitochondrial Primers (Mit1C), for the detection of cyclospora cayetanensis in produce. J Food Prot. 2023;86(2):100037. doi: 10.1016/j.jfp.2022.100037 36916572

[17] Temesgen TT, Tysnes KR, Robertson LJ. A new protocol for molecular detection of *Cyclospora cayetanensis* as contaminants of berry fruits. Front Microbiol. 2019;10:1939. doi: 10.3389/fmicb.2019.01939 31507557 PMC6719520

[18] Lalonde L, Oakley J, Fries P. Verification and use of the US-FDA BAM 19b method for detection of *Cyclospora cayetanensis* in a survey of fresh produce by CFIA laboratory. Microorganisms. 2022;10(3):559. doi: 10.3390/microorganisms10030559 35336134 PMC8954584

[19] U.S. Food and Drug Administration. Other analytical methods of interest to the foods program. https://www.fda.gov/food/laboratory-methods-food/other-analytical-methods-interest-foods-program. 2024. Accessed 2025 July 21.

[20] Deer DM, Lampel KA, González-Escalona N. A versatile internal control for use as DNA in real-time PCR and as RNA in real-time reverse transcription PCR assays. Lett Appl Microbiol. 2010;50(4):366–72. doi: 10.1111/j.1472-765X.2010.02804.x 20149084

[21] Kahler AM, Hofstetter J, Arrowood M, Peterson A, Jacobson D, Barratt J, et al. Sources and prevalence of *Cyclospora cayetanensis* in Southeastern U.S. Growing Environments. J Food Prot. 2024;87(7):100309. doi: 10.1016/j.jfp.2024.100309 38815808 PMC11288253

[22] Broeders S, Huber I, Grohmann L, Berben G, Taverniers I, Mazzara M, et al. Guidelines for validation of qualitative real-time PCR methods. Trends in Food Science & Technology. 2014;37(2):115–26. doi: 10.1016/j.tifs.2014.03.008

[23] Uhlig S, Frost K, Colson B, Simon K, Mäde D, Reiting R, et al. Validation of qualitative PCR methods on the basis of mathematical–statistical modelling of the probability of detection. Accred Qual Assur. 2015;20(2):75–83. doi: 10.1007/s00769-015-1112-9

[24] Durigan M, Patregnani E, Gopinath GR, Ewing-Peeples L, Lee C, Murphy HR, et al. Development of a molecular marker based on the mitochondrial genome for detection of *Cyclospora cayetanensis* in food and water samples. Microorganisms. 2022;10(9):1762. doi: 10.3390/microorganisms10091762 36144364 PMC9504131

[25] Kahler AM, Mattioli MC, da Silva AJ, Hill V. Detection of *Cyclospora cayetanensis* in produce irrigation and wash water using large-volume sampling techniques. Food and Waterborne Parasitology. 2021;22:e00110. doi: 10.1016/j.fawpar.2021.e00110 PMC7930117 33681488

