# Supplementary material for: Optimization of the Mit1C real-time PCR assay using Bio-Rad CFX96 for the detection of Cyclospora cayetanensis in agricultural waters
Source: PLoS One. 2026 Aug 12;21(8):e0355957. doi: 10.1371/journal.pone.0355957 (PMC13465831; doi:10.1371/journal.pone.0355957)
Supplement: Supplementary file 1 [file pone.0355957.s001.docx]

**S1 Table. Sensitivity of the Mit1C real-time PCR assay using Mit1AA synthetic fragments**

|  |  | **Mit1C Target** | | **IAC Target** | |
| --- | --- | --- | --- | --- | --- |
| **Mit1AA (co)** | **Replicates** | **Success** | **Cq** | **Success** | **Cq** |
| 20 | 12 | 12 | 35.19±0.39 | 12 | 25.15±0.10 |
| 10 | 12 | 12 | 36.27±0.56 | 12 | 25.11±0.11 |
| 5 | 12 | 12 | 37.16±0.60 | 12 | 25.05±0.06 |
| 2 | 12 | 11 | 38.44±0.78 | 12 | 25.06±0.08 |
| 1 | 12 | 5 | 38.89±1.15 | 12 | 25.07±0.06 |
| 0.1 | 12 | 0 | UND | 12 | 25.05±0.06 |
